# Omics-based ecosurveillance for the assessment of ecosystem function, health, and resilience

**DOI:** 10.1042/ETLS20210261

**Published:** 2022-04-11

**Authors:** David J. Beale, Oliver A.H. Jones, Utpal Bose, James A. Broadbent, Thomas K. Walsh, Jodie van de Kamp, Andrew Bissett

**Affiliations:** 1 Land and Water, Commonwealth Scientific and Industrial Research Organisation, Ecosciences Precinct, Dutton Park QLD 4102, Australia; 2 Australian Centre for Research on Separation Science (ACROSS), School of Science, RMIT University, Bundoora West Campus, PO Box 71, Bundoora, VIC 3083, Australia; 3 Agriculture and Food, Commonwealth Scientific and Industrial Research Organisation, Queensland Bioscience Precinct, St Lucia, QLD 4067, Australia; 4 Land and Water, Commonwealth Scientific and Industrial Research Organisation, Acton, ACT 2601, Australia; 5 Oceans and Atmosphere, Commonwealth Scientific and Industrial Research Organisation, Battery Point, TAS 7004, Australia

**Keywords:** environmental monitoring, genomics, metabolomics, proteomics, systems biology, transcriptomics

## Abstract

Current environmental monitoring efforts often focus on known, regulated contaminants ignoring the potential effects of unmeasured compounds and/or environmental factors. These specific, targeted approaches lack broader environmental information and understanding, hindering effective environmental management and policy. Switching to comprehensive, untargeted monitoring of contaminants, organism health, and environmental factors, such as nutrients, temperature, and pH, would provide more effective monitoring with a likely concomitant increase in environmental health. However, even this method would not capture subtle biochemical changes in organisms induced by chronic toxicant exposure. Ecosurveillance is the systematic collection, analysis, and interpretation of ecosystem health-related data that can address this knowledge gap and provide much-needed additional lines of evidence to environmental monitoring programs. Its use would therefore be of great benefit to environmental management and assessment. Unfortunately, the science of ‘ecosurveillance’, especially omics-based ecosurveillance is not well known. Here, we give an overview of this emerging area and show how it has been beneficially applied in a range of systems. We anticipate this review to be a starting point for further efforts to improve environmental monitoring via the integration of comprehensive chemical assessments and molecular biology-based approaches. Bringing multiple levels of omics technology-based assessment together into a systems-wide ecosurveillance approach will bring a greater understanding of the environment, particularly the microbial communities upon which we ultimately rely to remediate perturbed ecosystems.

## Introduction

**Figure 1.**
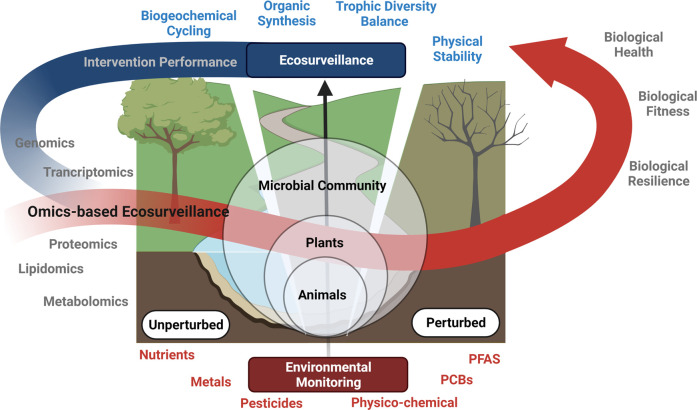
An overview of an ecosurveillance framework that encompasses omics-based technologies within a monitoring and surveillance setting. Note, PFAS, Perfluoroalkyl and Polyfluoroalkyl Substances; PCBs, Polychlorinated biphenyls.

Ecosurveillance, in the context of this review, is defined as the systematic collection, analysis, and interpretation of information on ecosystem health. It synthesises numerous data sources to inform environmental management and policy decisions. Environmental monitoring refers to the measurement of specific parameters, such as nutrients, dissolved oxygen, specific contaminants, and indicator organisms, usually concerning regulatory targets. Our ability to collect and interpret both ecosystem data and the inorganic/organic contaminant information represents a powerful tool for managing environmental health. Ecosurveillance can then also be used to measure the success or failure of interventions in a quantitative manner. This is graphically summarised in Figure 1.

The key point of differentiation between health/surveillance-based measurements of the ecosystem and monitoring of discrete and individual parameters/specific contaminants is that monitoring is more concerned with source-dictated (targeted) measurements (i.e. contaminants, nutrients, etc.) that are present in an environment, whereas ecosystem health metrics are focused on system-wide performance and characterisation (i.e. ecosystem service function/dysfunction due to biotic or abiotic perturbation). Omics-based technologies (i.e. genomics, transcriptomics, proteomics, lipidomics, and metabolomics) may be deployed within the ecosurveillance monitoring framework to capture the biological response of the system under perturbation.

## Environmental monitoring

A common approach for environmental monitoring is to take a targeted risk-based approach that considers known contaminant sources and associated industrial activities (both historical and current) in the catchment to compile a list of possible contaminants and physical parameters for monitoring [1]. The risk of this approach is that it can only monitor contaminants known to be present, it cannot identify unknown compounds or contaminants of concern that were not known to present, nor consider the cumulative effects of multiple sublethal stressors [2]. It is a static measurement of a fixed set of contaminants at a particular point in time, with no real link to the surrounding system outside of the matrix that was measured. Such assessments, therefore, do not indicate the bioavailability, stability, turnover, or distribution of the substances within the system. This can be a particular problem for contaminants such as perfluoroalkyl substances (PFAS), which are numerous (many undocumented), highly mobile, and are known to bioaccumulate in multiple trophic levels [3,4]; for long-lived contaminants that can bind to and release from sediments (e.g. metals); or, for contaminants that are formed in the system itself (e.g. photochemically produced reactive oxygen species) [5]. Furthermore, understanding the complex interactions between biological systems and anthropogenic environmental changes and the effects of natural variability in weather, season, and diurnal and biogeochemical cycles remains a major research challenge [6–8].

Traditional environmental and organism health monitoring techniques (e.g. chemical monitoring and bioassays) are highly suited to assessing acute toxic effects. They struggle, however, to detect subtle shifts in ecosystem function, species abundances, and animal physiology resulting from low-level and chronic exposures due to changing environmental/climatic conditions and interactions thereof.

Policy decisions are thus often based not on the actual health of the ecosystem, but the quantification of specific contaminants or nutrients above or below an arbitrary legislative level. To some degree, the importance of this gap between the measurement of a substance and the impact will vary with specific circumstances (e.g. season), but if the goal is a healthy and sustainable environment then lack of such information may hinder meaningful policy development and effective mitigation/harm reduction [9].

We suggest the data suitable for determining *in situ* risks of adverse effects or ecosystem perturbation are currently lacking for most environmental contaminants, both for prospective and retrospective assessments. Compounding this further is the uncertainty in ecological risk assessments of chemical mixtures (PFAS, pharmaceuticals, pesticides, chlorinated paraffins, and antibiotics are all cases in point) [10,11]. There is a clear need for better predictive tools for chemical mixture assessment, and the development of *in vitro*, and *in vivo* methods to efficiently assess and predict biological effects [12–14]. Effects Directed Analysis (EDA) is one approach to meeting this need, using metabolic sample fractionation, bioassay endpoints, and chemical contaminant analyses to identify key toxicants in the environment [15–17]. While EDA approaches are not the focus of this review, it is acknowledged that they would complement functional omics assays (i.e. metabolomics, lipidomics, and proteomics) used within the ecosurveillance framework proposed within that are aimed at capturing biological information from a sample matrix, which could be further analysed via the EDA pipeline of assays. Furthermore, novel non-targeted screening approaches that can capture contaminants outside of a risk-based monitoring framework have the potential to improve biological contaminant impact assessments and facilitate retrospective data mining for further insights into key contaminants of concern [18], if analysed with appropriately matched omics data (e.g. genomics, transcriptomics, proteomics, or metabolomics).

Ecosurveillance has the potential to bridge the gap in current monitoring data, and link system performance metrics that are tied to measured biotic and abiotic perturbations [19–21]. Omics-based approaches, either applied individually or in combination, have successfully been applied to investigate (potential or measured) function across the tree of life from microbial communities (i.e. microbiomes) through to larger metazoan taxa. Coupling measurable metabolic endpoints to multiple (sometimes subacute) contaminant levels and sources that are currently not identified using a conventional risk-based monitoring program has the potential to provide a more detailed and holistic understanding of temporal, spatial, and multigenerational impacts of contaminants on a single organism or species through to a whole-of-system approach [2]. For example, if one could measure metabolite levels and toxicant concentrations in the same analytical run there would be great potential for directly correlating metabolic changes and the contaminant concentrations needed to cause such changes. Even if contaminant levels were low, the presence of a contaminant, or more likely contaminants, known to cause a particular physiological change before significant harm occurring might at least be used as a trigger for further intensified monitoring and/or remediation before serious harm occurred.

The purpose of this review is to provide an overview of some of the salient applications of environmental functional omics within an omics-based ecosurveillance framework. We also demonstrate how these methods can be coupled with current monitoring data to better inform ecosystem health, function, and resilience.

### Environmental DNA (eDNA) and nucleic acid-based approaches as a monitoring tool

Environmental DNA (eDNA) methods focus on the detection and monitoring of sequenced nucleic acid data extracted from environmental samples and can be classed as a form of environmental genomics. They have been successfully used to survey fish biodiversity [22], invasive species [23], detect signature bacteria associated with contaminants [24], assess environmental health and status using indicator organisms [25,26], for species monitoring [23], exploring trophic interactions [27] through to investigating broad biogeographic patterns [28].

Principally, sequence-based eDNA data is a presence/absence or relative abundance measurement used for detection of organisms or function-specific genes (e.g. measuring nitrogen cycle genes in soils to monitor the effects of land-use changes [29]). The use of eDNA data for monitoring has largely focused on taxonomic observations of indicator organisms [30,31]. In the majority of cases, health and function are inferred from the presence/absence or relative abundance of these indicator organisms, with this data then used to inform a response or intervention [32]. Nevertheless, taxonomic observations do not necessarily inform us of the functional performance of an ecosystem. The function can be inferred from taxonomy where we have existing knowledge of an organism's functional capabilities or through bioinformatic functional inference approaches [33]. However, this is not always straightforward; this is particularly true in the case of microbial indicators. Microbiome metabolic capabilities can be decoupled from taxonomic identity (through gene loss, horizontal gene transfer [34]) and a single organism can exhibit a range of metabolic capabilities depending on environmental conditions [35].

Microbial communities drive key ecosystem processes in natural, managed, and engineered environments [36] and are intrinsically linked to the ecosystem state. Understanding microbiome processes that can mitigate environmental pollutants is therefore critical to maintaining rich biodiversity and healthy ecosystems [37]. Currently, existent and developing knowledge of how microbial communities function naturally and in response to perturbation is not well incorporated into environmental monitoring, surveillance, or management practices [38]. To a large extent, this extends up through producers and consumer taxa — as the interactions and levels of influence of ecosystem connectivity for many taxa are unknown.

Functional profiles of organisms or communities can be produced using DNA-based methods such as metabarcoding of functional genes in biochemical pathways or whole-metagenome sequencing (WMS) which has emerged as a powerful tool to survey biological community structures and their predicted function [39]. Additional analysis that complements and expand traditional metagenomic profiling and targeted eDNA metabarcoding techniques, capturing species-specific and microbial community functional activities are however, still needed [40].

DNA approaches, like those described above, target the genetic or functional *potential* of organisms or communities, not the realised functional activity or response. Efforts have been made to link gene and transcript abundances of genes associated with specific functions (e.g. ammonia oxidation, denitrification, etc.) with rates of activity, but success has been variable [41,42]. Transcriptomes, sequences of RNA from transcribed genes, take us a step closer to realised function by identifying active organisms and genetic pathways. The presence of specific transcripts does not, however, indicate the associated function is taking place since regulation can occur after expression [43]. Many enzymes also function to catalyse reactions in both directions, making it difficult to know the outcome of activity from gene/expression alone. Therefore, both DNA and RNA approaches are limited in that they do not represent the realised functional components of an environmental response. This is determined by the proteins translated from the RNA, transcribed from the DNA. The metabolome (metabolite content) is then the evidence of the protein activity. Proteomics and metabolomics used in an integrated ecosurveillance framework have the potential to improve our understanding of the realised ecosystem functional state, and with further development, mechanistic models of measurable health and function that can be used to improve management outcomes [23]. However, while DNA and RNA approaches have been readily accessible due to advances in technology for sequencing, quantifying, and comparing these data, until recently, proteomics and metabolomics lacked the depth and sensitivity to be useful except in very targeted experimental approaches. This is true of both more recently targeted organic metabolites (the focus of this review) and those inorganic metabolites we often consider readily measurable (e.g. the intermediates and end products of oxidative and reductive N processes), but which are very difficult to measure at appropriate temporal and spatial scales *in situ*. With the development of new technology and computational tools, however, applications outside of the laboratory setting are becoming more common.

### Monitoring function (metaproteome and metabolome)

Recent advances in (meta)proteomics and (community) metabolomics have provided a link between genomic expression and functional characterisation of ecosystem taxa [44]. This provides valuable insight into their *in situ* metabolism and function, under a range of biotic and abiotic perturbations that genomics alone cannot provide. Table 1 provides an overview of some recent omics-based ecosurveillance applications, which to date, have been predominately microbiome-based.

**Table 1:** Applications of ecosurveillance research with integrated functional datasets intended for omics-based ecosurveillance

Matrix	Objective	Omics				Reference
		Metagenomics	(Meta)transcriptomics	(Meta)proteomics	Metabolomics	
Soil	Measure the functional and phylogenetic responses of the microbial community impacted by drought.	• 16S rRNA gene (bacteria) • ITS (fungal)		• LC–MS/MS (Orbitrap)		Bastida et al. [45]
Soil	Detect active DNA viruses and RNA viruses in a native prairie soil and determine their responses to extremes in soil moisture	• 16S rRNA gene (bacteria)	• 16S rRNA gene	• Viral peptides LC–MS/MS		Wu et al. [46]
Soil	Assess the metaphenomic responses of a native prairie soil microbiome impacted by drought	• 16S rRNA gene (bacteria)	• 16S rRNA gene		• GC–MSD (Single quadrupole)	Roy Chowdhury et al. [47]
Soil	Assess microbial community compositions and functions in response to drought and rainfall events			• LC–MS/MS (Orbitrap)		Liu et al. [48]
Soil (microcosm)	Assessing organic matter decomposition and nutrient cycling in wetland soils	• 16S rRNA gene (bacteria)		• LC–MS/MS (Orbitrap)	• ^1^H NMR (600 MHz) • LC–MS/MS (Orbitrap)	McGivern et al. [49]
Soil	Assessing contaminants on agricultural microbiome metabolism	• 16S rRNA gene (bacteria) (after Xu et al. [50])		• LC–MS/MS	• LC–MS/MS	Chen et al. [51]
River	Assessment of surface water quality from multiple non-point source contaminants	• 16S rRNA gene (bacteria)			• GC–MSD (Single quadrupole)	Beale et al. [52]
Soil (-root interface)	Investigated the symbiotic associations between plant roots with rhizospheric bacterial communities under differing acid mine drainage pollution	• 16S rDNA gene (bacteria)			• LC-TQ-MS	Kalu et al. [53]
Soil	Responses of soil microorganisms to polycyclic aromatic hydrocarbon stress	• 16S rDNA gene (bacteria) with functional prediction of genes (PICRUSt)			• GC-QToF-MS	Li et al. [54]
Sediment/Water (microcosm)	Response of indigenous microbial structure and functional dynamics in different marine environmental matrices after oil exposure	• 16S rDNA gene (bacteria) with functional prediction of genes (PICRUSt)			• Predicted from PICRUSt	Neethu et al. [55]
Marine Sediment	Measure the influence of estuarine macrophytes on sediment microbial function and metabolic redundancy	• 16S rRNA gene (bacteria) with functional prediction of genes (PICRUSt)			• LC-TQ-MS • LC-QToF-MS	Shah et al. [56]
River (flumes)	Impact of sulfamethoxazole on a riverine microbiome	• 16S rRNA gene (bacteria)		• LC–MS/MS (Orbitrap)		Borsetto et al. [57]
Marine	Microbial processing of organic matter throughout the water column	• 16S rRNA gene (bacteria)		• LC-TQ-MS		Bergauer et al. [58]
Soil	Investigating agroecosystem microbial community strategies during low water availability	• 16S rDNA gene (bacteria)		• LC–MS/MS		Starke et al. [59]
Marine Sediment	Investigated microbial methane oxidation at the sediment–water interface of a shallow marine methane seep	• 16S rRNA gene (bacteria)		• Stable isotope probing (SIP) LC–MS/MS (Orbitrap)		Taubert et al. [60]
Permafrost	Reconstruction of fossil and living microorganisms in ancient permafrost	• 16S rDNA gene (bacteria)		• LC-TQ-MS		Liang et al. [61]
Sediment	Measuring the kinetics of biogeochemical processes in natural and engineered environmental systems	• 16S rRNA gene (bacteria and archaea)		• LC-TQ-MS		Li et al. [62]
Soil	Investigated synergistic interactions in a bisphenol A (BPA)-degrading microbial community	• 16S rDNA gene (bacteria)	• 16S rRNA-tag pyrosequencing			Yu et al. [63]
Sediment (microcosm)	Elucidate the mechanisms driving the rapid biodegradation of Deepwater Horizon Oil in intertidal sediments	• 16S rDNA gene (bacteria)	• 16S rRNA			Karthikeyan et al. [64]
Soil	Investigate soil fungi and their relation to edaphic and environmental variables across three ecosystems	• 18S rRNA gene		• LC–MS/MS (Orbitrap)		Fernandes et al. [65]
Sediment	Measure the biological impacts across multiple trophic levels of offshore oil and gas drilling and production operations	• 16S rDNA (bacteria) • 18S rDNA (eukaryotes) • 18S rDNA (foraminifera)	• 16S rRNA (bacteria) • 18S rRNA (eukaryotes) • 18S rRNA (foraminifera)			Laroche et al. [66]

#### Proteomics and metaproteomics applications

Proteomics has been applied to investigate and monitor the effect of abiotic factors in selected microbiomes, wild species, and model organisms; however, its application to broader ecological monitoring remains underdeveloped. Where a proteomic investigation has been applied to abiotic stress models, much focus has been placed on contaminated microbiomes [67,68] and aquatic species such as fish, crustaceans, and molluscs [69]. As an example, the proteome of goldfish (*Carassius auratus*) was shown to produce significant changes to oxidative stress and apoptosis inhibition, with no mortality, when subjected to herbicide and fungicide mixtures (8.4 and 42 μg L^−1^, respectively) and high temperatures (22 and 32°C) [70]. Likewise, the exposure of mature male and female White Sucker fish (*Catostomus commersonii*) to oil sands-related chemicals in the Athabasca River in Canada showed altered lipid and endocrine metabolism perturbations [71]. The exposure to two different doses of commercial herbicide led to growth impairment and perturbation of the hepatic proteome of rainbow trout (*Oncorhynchus mykiss*) [72].

Continuous exposure to environmental pollutants has been shown to damage the redox response and detoxification processes in crustaceans [73]. Bivalves exposed to metals such as cadmium, copper, lead, and zinc have shown an increased abundance of proteins associated with stress responses, cytoskeletal activity, and protein synthesis [74]. A recent multi-omics study from our group, investigating freshwater turtles exposed to PFAS, identified signs of elevated immune activity and perturbed lipid transport and binding [3]. In addition to monitoring the aquatic environment, transcriptomic- and proteomic-based experiments have provided evidence that herbicides can affect life cycle mechanisms including moulting and the reproduction process of the springtail *Folsomia candida* [75]. Each of these studies presents excellent knowledge development in the assessment of abiotic stress impact on environmental samples or models, but the transition of this knowledge to practical ecosurveillance application remains unfulfilled.

The expansion of metaproteomic approaches that are targeted towards the study of how microbes contribute to ecosystem services [76], capturing both phylogenetic and functional information would help address this. Environmental microbiomes are highly diverse but are currently largely under-represented in public proteomics databases. Furthermore, functional characterisation using metaproteomics is usually performed with the aid of metagenomic sequences acquired for the same sample. Truly representative and diverse metagenomic datasets are difficult to assemble, and therefore, the utility of existing high-quality theoretical proteome databases covering many known isolates eliminating the need for sequencing, is often preferred [77]. A recent proteome-wide study conducted on the taxonomy of life has shown that even after using organism-specific genome and transcriptome resources, ∼40% of the identified proteins did not have any functional annotation for their biological processes [78]. Thus, the success of eco-omics-based studies will require improvement in proteome and metaproteome analysis which can reveal the over-representation of functional classes to obtain a global view of environmental health rather than a species-specific view.

Analysis of complex environments is often hindered by the heterogeneity of the sample matrix and its varying concentrations of interfering substances (i.e. salts, humic, fulvic, and tannic acids) [79] that can negatively impact extraction and recovery efficiencies [44,80]. Commercial kits are currently available for the co-extraction of DNA and RNA, but the inclusion of proteins and metabolites requires more research and development [81,82].

#### Metabolomics and community metabolomics applications

Metabolomics is well suited to assess sublethal biological effects of contaminants and chemical mixtures; it relates chemical processes, intermediates, and end-products of an organism's metabolism and is closely linked to an exposure-induced phenotype. Coupling quantitative and qualitative chemical analyses with environmental metabolomics bioassays for a range of species/ages/sexes/developmental stages, as is currently underway, will be particularly pertinent in establishing omics-based models to understand the contaminant exposure and impact pathways. For example, metabolomics has recently been applied to zebrafish exposed to environmentally relevant levels of climbazole, a topical antifungal agent, to elucidate the biochemical reasons for reproductive abnormalities seen in female fish [83]. Metabolomics methods have been used to elucidate the sublethal effects of toxicants on a large range of species [84–88].

Where environmental metabolomics comes to the forefront in ecosurveillance applications is with its application to deep data science investigations, coupled with monitoring metadata and other omics datasets for investigating ecosystem homeostasis [89]. For example, metabolomics has been used to identify correlations between the root microbiome and plant gene translocation in varying plant functions when perturbed by mine drainage pollution that ultimately improved its adaptability and phytoremediation potential [53]. Integrative analyses of transcriptomes and metabolomes in microalgae (*Raphidocelis subcapitata*) treated with the antibiotic clarithromycin [90] identified impacts to biosynthesis and photosynthesis, highlighting the inhibitory effects of macrolide antibiotics.

Of particular interest is our current understanding of microbial function within environmental microbiomes, which stems from conventional ecology-based surveys and the utility of more recent environmental genomics approaches (eDNA). Attempts to harmonise these data with physicochemical parameters of biotic/abiotic ecosystem (dys)function arising from environmental metabolomics data have been limited and show varying levels of success [9,21,52]. This is potentially biased towards a limited group of microbes (e.g. by the choice of primers), a selection of discrete sampling points, and/or failure to link microbial diversity with functions related to biogeochemical cycles or well-defined metabolic endpoints [48]. So, while resilience and redundancy are theoretically assessed (via*,* for example, amplicon sequencing), an actual measurable function that is quantifiable is often not. More recent research by Shah et al. [91] employed phylogenetic reconstruction methods to infer genome content and predict functional (relative) abundances that can be matched to metabolite features that are either expressed or consumed within the analysed microbiome.

Like metaproteomic approaches, community metabolomics (e.g. metabolomics of entire communities, usually microbial) may provide functional information post enrichment without the need for 16S rRNA community diversity profiles. Currently, this method has been largely limited to use in soil [92,93] and some specific aquatic systems [52,94]. Just as eDNA can be amplified and used to give an idea of what organisms are present in aquatic systems, so could an environment's community metabolic profile be preconcentrated, cleaned-up (e.g. via solid-phase extraction), and measured in large aquatic systems such as lakes and streams. These data will generate information on the environmental metabolome and the impact of environmental perturbations on the community. It may also elucidate specific functions such as host-pathogen interactions and plant signalling compounds, and associated responses that are aggregated over physicochemical cycles (i.e. spring-neap tide cycles, etc.) [56,91].

One potential weakness of metabolomics is that results tend to represent the situation at a particular point in time (when the samples were taken). Organisms, however, exist in time and changes are dependent on developmental stage, and external factors, such as climate and health or symbiotic relationship, that can affect an organism's susceptibility to a pollutant (or pollutants) and thus its potential risk. For it to be a useful tool for monitoring, we need environmental metabolomics to help us understand how differences in timing and duration of exposure (and subsequent depuration) influence metabolite profiles and organism health. This could be achieved through long-term multi-generational studies, short-term diurnal flux sampling, or by combining existing metabolomics data from model species in the literature and studies performed on the same organism with the same pollutant, but at different life-history stages to give a more comprehensive overview of effects. The integration of *in vitro* metabolomics with high-throughput screening platforms such as those recently demonstrated by Malinowska et al. [95] may also help with this aim.

## Integrating multi-omic datasets for ecosurveillance

The expansion of multi-omics research has driven the development of new tools and web-based applications that facilitate their integration for deeper interpretations that extend beyond the correlation of biological molecular features towards biological causality and response. Recent expansion, and a renewed focus on the human gut microbiome, has also led to the development of microbiome-centric tools that pair microbiome sequencing (16S rRNA gene amplicons, shotgun metagenomics, and metatranscriptomics) datasets with analysed metabolomes (mass spectrometry and nuclear magnetic resonance spectroscopy data) [96]. While some of these tools have predominantly been driven by medical and clinical research (e.g. MIMOSA2), they have proven utility when applied to environmental datasets and omics-based ecosurveillance.

Examples of these ‘*clinical*’ tools being applied within an environmental context are growing; Hua et al. [97] applied MIMOSA2 to investigate the gastrointestinal microbiome of zebrafish (*Danio rerio*) exposed to the organochlorine pesticide dieldrin, coupling sequence datasets with measure metabolite data. Shah et al. coupled 16S rRNA gene sequencing datasets with untargeted metabolomics data to assess the function of marine sediments in tropical estuaries [91] and differing macrophyte zones [56]. Table 2 provides a summary of the recent tools that are freely available; a more expansive list is provided in the review by Pinu et al. [98].

**Table 2:** Tools and applications for integrating multi-omics datasets intended for omics-based ecosurveillance

Tool	Source	Description	Data inputs	Reference
Web of microbes	http://webofmicrobes.org	Web-based exometabolomics data repository and visualization tool.	• None. Data mining tool.	Kosina et al. [99]
MIMOSA2	http://elbo-spice.cs.tau.ac.il/shiny/MIMOSA2shiny/	Web-based and R-based metabolic network tool for inferring mechanism-supported relationships in microbiome-metabolome datasets.	• Taxonomic and/or functional abundances • Metabolite data table (KEGG or HMDB)	Noecker et al. [100]
MelonnPan	http://huttenhower.sph.harvard.edu/melonnpan.	R-based tool for computational framework modelling to predict community metabolomes from microbial community profiles.	• Taxonomic and/or functional abundances • Metabolite data table	Mallick et al. [101]
MicrobiomeAnlayst	https://www.microbiomeanalyst.ca/	Web-based tool for the comprehensive analysis of common data outputs generated from microbiome studies. Provides a prediction of function based on species annotations.	• Taxonomic and/or functional abundances	Chong et al. [102]
Reactome	https://reactome.org/	Web-based multi-omics data visualization and metabolic mapping tool of known biological processes and pathways	• Multi-omics datasets (multiple common formats)	Griss et al. [103]
PaintOmics 3.0	http://www.paintomics.org/	Web-based tool for the joint visualization of genomics/transcriptomics, proteomics, and metabolomics data.	• Multi-omics datasets (multiple common formats)	Hernández-de-Diego et al. [104]
mixOmics	http://mixomics.org/	An R-based multivariate tool that is suited to large ‘omics data sets where the number of variables (e.g. genes, proteins, metabolites) is much larger than the number of samples.	• Transcriptomics, metabolomics, proteomics, microbiome/metagenomics	Rohart et al. [105]
OmicsAnalyst	https://www.omicsanalyst.ca/	Web-based data-driven multi-omics integration tool via intuitive visual analytics	• Transcriptomics, proteomics, metabolomics, and miRNA data	Zhou et al. [106]
OmicsNet 2.0	https://www.omicsnet.ca/OmicsNet/home.xhtml	Web-based data-driven multi-omics integration tool via Knowledge-based networks	• Transcriptomics, proteomics, metabolomics, and miRNA data	Zhou and Xia [107]

It is now viable to couple ecosurveillance and monitoring data within a multi-omics framework, with computational methods to create novel and integrated ecosystem-scale data frames of system function, organism health, and ecological productivity. This would highlight links between different taxonomic levels, pulling together common metabolic features (harmonisation) into a unified predictive model of ecosystem service provision and system health/trajectory. Such an approach that harmonises conserved metabolic traits amongst taxa with emergent properties of concern (i.e. chemicals and physical attributes), identified via non-target screening, could be utilised in ecosystem predictive models to improve management intervention opportunities, thereby enabling better management decisions and policies.

## Concluding remarks and future perspectives

A critical mass in genomic resources, analytical technologies, and bioinformatics approaches has provided unprecedented insights into the composition, structure, function, and control of the genome, transcriptome, proteome, and metabolome, shedding light upon numerous known and unknown biological pathways and phenotypes. Though omics technologies are constantly being adapted to ecological research, there are still limitations and challenges that need to be considered to harness their full potential and acceptance by industry and regulators. In particular, for the application of proteomics to ecological monitoring, the primary issues such as sample and genetic heterogeneity, and limited genomic resources for non-model species complicate data interpretation and limit the potential for integration with other ‘*omes’* to obtain systems-level information [108]. Improvement of sampling techniques such as ‘*single-pot’* sample extractions could be useful in ensuring the use of a single sample (tube) for multiple omic measurements for a more resolved data interpretation. Additionally, *de novo* genome sequencing and species-specific database construction would be valuable to identify and validate markers for monitoring purposes. The consideration of *intra-* and *inter-*species population diversity, genetic polymorphism, phenotypic plasticity, and developmental stages (including the alternative splicing, polypeptide cleavage, post-translational modification) should also be taken into account to inform proteome measurements and other ‘omics-based outputs to better understand taxonomically similar species on a system level [109].

Another area that will likely advance in the future is metabolite identification [110]. At present, many of what is labelled as ‘*features’* in metabolomics datasets are not identified [111]. This limits our potential gain in knowledge and understanding of environmental processes. However, collecting robust environmental metadata around these unidentified features could allow them to be correlated and characterised into an environmental context, even if we don't know their specific function (e.g. metabolite feature X always occurs within Y environments with high metal loads, etc.).

There is currently a paucity of proteome sequence databases available for non-model species. The incompleteness of sequence databases and their limited annotations are often considered a bottleneck for environmental proteomics experiments. Thus, the precise assembly and annotation of genomic and transcriptomic resources would be key to decoding and monitoring proteome level changes in non-model species and their relationship to environmental health. Similar limitations occur in metabolomics. At this point, two main strategies for dealing with metabolomics datasets (which tend to be very large) have involved (1) the establishment of spectral databases to aid with individual feature identification, e.g. the Human Metabolome Database (www.hmdb.ca) [112] and METLIN (https://metlin.scripps.edu/) [113], and (2) developing workflows and analytical packages to facilitate multivariate statistics on individual experimental outcomes. The creation of interactive and open-access databases of pollutants such as the toxic exposome database (http://www.t3db.ca) [114], DrugBank (https://go.drugbank.com) [115], and the EPA's non-targeted analysis (NTA) database [116] have helped, but each lists data on contaminants/toxicants, not the metabolic response(s) to such compounds. What would help in the future is a library of metabolite profiles for model species exposed to specific pollutants, or mixtures of pollutants as a dedicated tool to facilitate environmental monitoring in complex aquatic environments. The knowledge and infrastructure from existing metabolomics databases could be used for data management, interactive storage, and access to such a system, but it would be reliant on high-quality data from the community to function. Such a database, that is publicly available and easily searchable would facilitate the use of metabolomics in environmental science by allowing scientists to compare results of the analysis of a system, to the metabolic response(s) of the organisms to known pollutants/toxicants (further building upon the ‘Web of microbes’ exometabolomics database for linking chemistry and microbes [99]).

Once a comprehensive database is available, data acquisition techniques such as data-independent acquisition (DIA)-based approaches could be applied to monitor continuous changes. These label-free proteome measurement approaches bring the best of shotgun and targeted acquisition, allowing deep proteome analysis and accurate and reproducible quantitation of proteomes [117], including meta-proteomes [118]. This has recently been supported by advanced machine learning-based tools such as the DIA-NN software [119], which uses neural networks to determine ‘*real signals*’ from the noise for quantitation and interference removal without any retention time alignment and also makes use of the Prosit [120] ML predictor to prepare synthetic peptide spectral libraries for quantifying thousands of proteins without a previous observation being required.

Multiple levels of omics-based data coupled within an ecosurveillance approach will bring a greater understanding of the natural and perturbed environment, particularly the microbial systems, upon which we ultimately rely to remediate contaminants. This will be of great benefit to human and environmental health. However, for these multi-omic studies to be conducted in parallel with current approaches to demonstrate their value-add to the *status-quo*, regulators and funding models need to account for the perceived inherent risk of trialling these new approaches (and allow for financial mechanisms that cover the additional analytical costs and help to carry any regulatory risk of just an omics-only approach). Furthermore, agencies must allow for open-ended studies that include (or rely on) nontargeted data but also encompass repeat non-target/omics measures over time (i.e. monthly and yearly) that can seem very open-ended to a regulator. It is only then that multi-omics guided ecosurveillance could demonstrate a pathway for improved management intervention opportunities, management decisions, and policies since there are currently limited practical examples of these tools guiding these processes and decisions within the environmental regulatory framework.

In closing, we encourage all readers to explore the opportunities in this exciting area of omics-based research, and as a community of researchers and practitioners, to demonstrate their value and keep pushing these approaches until they become part of the regulatory framework and are embedded in ecosystems monitoring programs.

## Summary

- Current environmental monitoring efforts often focus on known, regulated contaminants ignoring the effects of unmeasured compounds, environmental factors, and subtle biochemical perturbations.
- Metabolomics- and proteomics-based approaches can be coupled with DNA/RNA sequence technologies to provide measured functional outputs.
- Using multiple levels of omics technology-based assessments together into an ecosurveillance approach will bring a greater understanding of the natural and perturbed environment with great benefit to environmental health.

## Open Access

Open access for this article was enabled by the participation of CSIRO in an all-inclusive *Read & Publish* agreement with Portland Press and the Biochemical Society under a transformative agreement with CAUL.

## Abbreviations

DIA: data-independent acquisition
EDA: effects directed analysis
PFAS: perfluoroalkyl substances

## References

[1] ANZECC and ARMCANZ. (2018) Australian and New Zealand Guidelines for Fresh and Marine Water Qualit. [cited 2021; Available from: https://www.waterquality.gov.au/anz-guidelines

[2] Ebner, J.N. (2021) Trends in the application of “omics” to ecotoxicology and stress ecology. Genes 12, 1481 10.3390/genes12101481 34680873 PMC8535992

[3] Beale, D.J., Hillyer, K., Nilsson, S., Limpus, D., Bose, U., Broadbent, J.A. et al. (2022) Bioaccumulation and metabolic response of PFAS mixtures in wild-caught freshwater turtles (Emydura macquariimacquarii) using omics-based ecosurveillance techniques. Sci. Total Environ. 806, 151264 10.1016/j.scitotenv.2021.151264 34715216

[4] Beale, D.J., Nilsson, S., Bose, U., Bourne, N., Stockwell, S., Broadbent, J.A. et al. (2022) Bioaccumulation and impact of maternal PFAS offloading on egg biochemistry from wild-caught freshwater turtles (Emydura macquarii macquarii). Sci. Total Environ. 817, 153019 10.1016/j.scitotenv.2022.153019 35026273

[5] Adesina, A.O., Anifowose, A.J., Takeda, K. and Sakugawa, H. (2018) Photogeneration and interactive reactions of three reactive species in the Seto Inland Sea, Japan. Environ. Chem. 15, 236–245 10.1071/EN18035

[6] Carrier-Belleau, C., Drolet, D., McKindsey, C.W. and Archambault, P. (2021) Environmental stressors, complex interactions and marine benthic communities’ responses. Sci. Rep. 11, 4194 10.1038/s41598-021-83533-1 33603048 PMC7892560

[7] Xia, J., Wang, J. and Niu, S. (2020) Research challenges and opportunities for using big data in global change biology. Glob. Chang. Biol. 26, 6040–6061 10.1111/gcb.15317 32799353

[8] Ebner, J.N., Ritz, D. and von Fumetti, S. (2020) Abiotic and past climatic conditions drive protein abundance variation among natural populations of the caddisfly crunoecia irrorata. Sci. Rep. 10, 15538 10.1038/s41598-020-72569-4 32968134 PMC7512004

[9] Shah, R.M., Crosswell, J., Metcalfe, S.S., Carlin, G., Morrison, P.D., Karpe, A.V. et al. (2019) Influence of human activities on broad-scale estuarine-marine habitats using omics-Based approaches applied to marine sediments. Microorganisms 7, 419 10.3390/microorganisms7100419 PMC6843362 31590307

[10] Drakvik, E., Altenburger, R., Aoki, Y., Backhaus, T., Bahadori, T., Barouki, R. et al. (2020) Statement on advancing the assessment of chemical mixtures and their risks for human health and the environment. Environ. Int. 134, 105267 10.1016/j.envint.2019.105267 31704565 PMC6979318

[11] Rodgers, K., McLellan, I., Peshkur, T., Williams, R., Tonner, R., Hursthouse, A.S. et al. (2019) Can the legacy of industrial pollution influence antimicrobial resistance in estuarine sediments? Environ. Chem. Lett. 17, 595–607 10.1007/s10311-018-0791-y

[12] Ankley, G.T., Cureton, P., Hoke, R.A., Houde, M., Kumar, A., Kurias, J. et al. (2021) Assessing the ecological risks of per- and polyfluoroalkyl substances: current state-of-the science and a proposed path forward. Environ. Toxicol. Chem. 40, 564–605 10.1002/etc.4869 32897586 PMC7984443

[13] Hernández-Mesa, M., Le Bizec, B. and Dervilly, G. (2021) Metabolomics in chemical risk analysis – A review. Anal. Chim. Acta 1154, 338298 10.1016/j.aca.2021.338298 33736812

[14] Proença, S., Escher, B.I., Fischer, F.C., Fisher, C., Grégoire, S., Hewitt, N.J. et al. (2021) Effective exposure of chemicals in in vitro cell systems: a review of chemical distribution models. Toxicol. in Vitro 73, 105133 10.1016/j.tiv.2021.105133 33662518

[15] Brack, W., Ait-Aissa, S., Burgess, R.M., Busch, W., Creusot, N., Di Paolo, C. et al. (2016) Effect-directed analysis supporting monitoring of aquatic environments — An in-depth overview. Sci. Total Environ. 544, 1073–1118 10.1016/j.scitotenv.2015.11.102 26779957

[16] Burgess, R.M., Ho, K.T., Brack, W. and Lamoree, M. (2013) Effects-directed analysis (EDA) and toxicity identification evaluation (TIE): complementary but different approaches for diagnosing causes of environmental toxicity. Environ. Toxicol. Chem. 32, 1935–1945 10.1002/etc.2299 23893495

[17] Martins, C., Dreij, K. and Costa, P.M. (2019) The state-of-the Art of environmental toxicogenomics: challenges and perspectives of “Omics” approaches directed to toxicant mixtures. Int. J. Environ. Res. Public Health 16, 4718 10.3390/ijerph16234718 PMC6926496 31779274

[18] Crimmins, B.S. and Holsen, T.M. (2019) Non-targeted Screening in Environmental Monitoring Programs. In Advancements of Mass Spectrometry in Biomedical Research (Woods, A.G. and Darie, C.C., eds), pp. 731–741, Springer International Publishing, Cham 10.1007/978-3-030-15950-4_43 31347081

[19] Zhang, A.-N., Gaston, J.M., Dai, C.L., Zhao, S., Poyet, M., Groussin, M. et al. (2021) An omics-based framework for assessing the health risk of antimicrobial resistance genes. Nat. Commun. 12, 4765 10.1038/s41467-021-25096-3 34362925 PMC8346589

[20] Bahamonde, P.A., Feswick, A., Isaacs, M.A., Munkittrick, K.R. and Martyniuk, C.J. (2016) Defining the role of omics in assessing ecosystem health: perspectives from the Canadian environmental monitoring program. Environ. Toxicol. Chem. 35, 20–35 10.1002/etc.3218 26771350

[21] Beale, D.J., Crosswell, J., Karpe, A.V., Ahmed, W., Williams, M., Morrison, P.D. et al. (2017) A multi-omics based ecological analysis of coastal marine sediments from gladstone, in Australia's central queensland, and heron island, a nearby fringing platform reef. Sci. Total Environ. 609, 842–853 10.1016/j.scitotenv.2017.07.184 28768216

[22] Xiong, F., Shu, L., Gan, X., Zeng, H., He, S. and Peng, Z. (2022) Methodology for fish biodiversity monitoring with environmental DNA metabarcoding: the primers, databases and bioinformatic pipelines. Water Biol. Security 1, 100007 10.1016/j.watbs.2022.100007

[23] Geist, J.A., Mancuso, J.L., Morin, M.M., Bommarito, K.P., Bovee, E.N., Wendell, D. et al. (2022) The New Zealand mud snail (Potamopyrgus antipodarum): autecology and management of a global invader. Biol. Invasions **24**, 905–938 10.1007/s10530-021-02681-7

[24] Abas, A.H., Marfuah, S., Abram, A.A.D.P., Kolondam, B.J. and Tallei, T.E. (2022) Environmental DNA (e-DNA) as a method for early detection of diesel Oil pollution: a review. J. Biotechnol. Conserv. Wallacea 1, 57–65

[25] Aylagas, E., Borja, Á., Tangherlini, M., Dell'Anno, A., Corinaldesi, C., Michell, C.T. et al. (2017) A bacterial community-based index to assess the ecological status of estuarine and coastal environments. Mar. Pollut. Bull. 114, 679–688 10.1016/j.marpolbul.2016.10.050 27784536

[26] Martínez, E.A. (2017) DNA metabarcoding derived biotic indices for marine monitoring and assessment

[27] Banerjee, P., Stewart, K.A., Antognazza, C.M., Bunholi, I.V., Deiner, K., Barnes, M.A. et al. (2021) Plant-animal interactions in the era of environmental DNA (eDNA)–a review. Authorea 10.22541/au.162626116.66217318/v1

[28] Gaither, M.R., DiBattista, J.D., Leray, M. and von der Heyden, S. (2021) Metabarcoding the marine environment: from single species to biogeographic patterns. Environ. DNA 4, 3–8 10.1002/edn3.270

[29] Colloff, M.J., Wakelin, S.A., Gomez, D. and Rogers, S.L. (2008) Detection of nitrogen cycle genes in soils for measuring the effects of changes in land use and management. Soil Biol. Biochem. 40, 1637–1645 10.1016/j.soilbio.2008.01.019

[30] Apothéloz-Perret-Gentil, L., Bouchez, A., Cordier, T., Cordonier, A., Guéguen, J., Rimet, F. et al. (2021) Monitoring the ecological status of rivers with diatom eDNA metabarcoding: a comparison of taxonomic markers and analytical approaches for the inference of a molecular diatom index. Mol. Ecol. 30, 2959–2968 10.1111/mec.15646 32979002 PMC8358953

[31] Aylagas, E., Atalah, J., Sánchez-Jerez, P., Pearman, J.K., Casado, N., Asensi, J. et al. (2021) A step towards the validation of bacteria biotic indices using DNA metabarcoding for benthic monitoring. Mol. Ecol. Resour. 21, 1889–1903 10.1111/1755-0998.13395 33825307

[32] Phiri, B.J., Hayman, D.T.S., Biggs, P.J., French, N.P. and Garcia-R, J.C. (2021) Microbial diversity in water and animal faeces: a metagenomic analysis to assess public health risk. N. Z. J. Zool. 48, 188–201 10.1080/03014223.2020.1831556

[33] Laroche, O., Pochon, X., Wood, S.A. and Keeley, N. (2021) Beyond taxonomy: validating functional inference approaches in the context of fish-farm impact assessments. Mol. Ecol. Resour. 21, 2264–2277 10.1111/1755-0998.13426 33971078

[34] Martiny, A.C., Treseder, K. and Pusch, G. (2013) Phylogenetic conservatism of functional traits in microorganisms. ISME J. 7, 830–838 10.1038/ismej.2012.160 23235290 PMC3603392

[35] Chen, Y.-J., Leung, P.M., Wood, J.L., Bay, S.K., Hugenholtz, P., Kessler, A.J. et al. (2021) Metabolic flexibility allows bacterial habitat generalists to become dominant in a frequently disturbed ecosystem. ISME J. 15, 2986–3004 10.1038/s41396-021-00988-w 33941890 PMC8443593

[36] Konopka, A. (2009) What is microbial community ecology? ISME J. 3, 1223–1230 10.1038/ismej.2009.88 19657372

[37] Barkay, T. and Pritchard, H. (1988) Adaptation of aquatic microbial communities to pollutant stress. Microbiol. Sci. 5, 165–169 PMID:3079233

[38] Banfield, J.F., Verberkmoes, N.C., Hettich, R.L. and Thelen, M.P. (2005) Proteogenomic approaches for the molecular characterization of natural microbial communities. Omics 9, 301–333 10.1089/omi.2005.9.301 16402891

[39] Wallace, J.C., Youngblood, J.E., Port, J.A., Cullen, A.C., Smith, M.N., Workman, T. et al. (2018) Variability in metagenomic samples from the puget sound: relationship to temporal and anthropogenic impacts. PLoS ONE 13, e0192412 10.1371/journal.pone.0192412 29438385 PMC5811002

[40] Franzosa, E.A., Hsu, T., Sirota-Madi, A., Shafquat, A., Abu-Ali, G., Morgan, X.C. et al. (2015) Sequencing and beyond: integrating molecular ‘omics’ for microbial community profiling. Nat. Rev. Microbiol. 13, 360–372 10.1038/nrmicro3451 25915636 PMC4800835

[41] Graham, E.B., Knelman, J.E., Schindlbacher, A., Siciliano, S., Breulmann, M., Yannarell, A. et al. (2016) Microbes as engines of ecosystem function: when does community structure enhance predictions of ecosystem processes? Front. Microbiol. 7, 214 10.3389/fmicb.2016.00214 26941732 PMC4764795

[42] Raes, E.J., Karsh, K., Kessler, A.J., Cook, P.L.M., Holmes, B.H., van de Kamp, J. et al. (2020) Can we use functional genetics to predict the fate of nitrogen in estuaries? Front. Microbiol. 11, 1261 10.3389/fmicb.2020.01261 32655525 PMC7325967

[43] Tsujimura, A., Yasojima, K., Kuboki, Y., Suzuki, A., Ueno, N., Shiokawa, K. et al. (1995) Developmental and differential regulations in gene expression of xenopus pleiotropic factors-α and -β. Biochem. Biophys. Res. Commun. 214, 432–439 10.1006/bbrc.1995.2305 7677748

[44] Russo, D.A., Couto, N., Beckerman, A.P. and Pandhal, J. (2019) Metaproteomics of freshwater microbial communities. Methods Mol. Biol. 1977, 145–155 10.1007/978-1-4939-9232-4_10 30980327

[45] Bastida, F., Torres, I.F., Andrés-Abellán, M., Baldrian, P., López-Mondéjar, R., Větrovský, T. et al. (2017) Differential sensitivity of total and active soil microbial communities to drought and forest management. Glob. Chang. Biol. 23, 4185–4203 10.1111/gcb.13790 28614633

[46] Wu, R., Davison, M.R., Gao, Y., Nicora, C.D., McDermott, J.E., Burnum-Johnson, K.E. et al. (2021) Moisture modulates soil reservoirs of active DNA and RNA viruses. Commun. Biol. 4, 992 10.1038/s42003-021-02514-2 34446837 PMC8390657

[47] Roy Chowdhury, T., Lee, J.-Y., Bottos, E.M., Brislawn, C.J., White, III, R.A., Bramer, L.M. et al. (2019) Metaphenomic responses of a native prairie soil microbiome to moisture perturbations. mSystems 4, e00061-19 10.1128/mSystems.00061-19 31186334 PMC6561317

[48] Liu, D., Keiblinger, K.M., Leitner, S., Wegner, U., Zimmermann, M., Fuchs, S. et al. (2019) Response of microbial communities and their metabolic functions to drying-Rewetting stress in a temperate forest soil. Microorganisms 7, 129 10.3390/microorganisms7050129 PMC6560457 31086038

[49] McGivern, B.B., Tfaily, M.M., Borton, M.A., Kosina, S.M., Daly, R.A., Nicora, C.D. et al. (2021) Decrypting bacterial polyphenol metabolism in an anoxic wetland soil. Nat. Commun. 12, 2466–2466 10.1038/s41467-021-22765-1 33927199 PMC8084988

[50] Xu, W., You, Y., Wang, Z., Chen, W., Zeng, J., Zhao, X. et al. (2018) Dibutyl phthalate alters the metabolic pathways of microbes in black soils. Sci. Rep. 8, 2605 10.1038/s41598-018-21030-8 29422490 PMC5805725

[51] Chen, W., Wang, Z., Xu, W., Tian, R. and Zeng, J. (2020) Dibutyl phthalate contamination accelerates the uptake and metabolism of sugars by microbes in black soil. Environ. Pollut. 262, 114332 10.1016/j.envpol.2020.114332 32182534

[52] Beale, D.J., Karpe, A.V., Ahmed, W., Cook, S., Morrison, P.D., Staley, C. et al. (2017) A community multi-Omics approach towards the assessment of surface water quality in an urban river system. Int. J. Environ. Res. Public Health 14, 303 10.3390/ijerph14030303 PMC5369139 28335448

[53] Kalu, C.M., Ogola, H.J.O., Selvarajan, R., Tekere, M. and Ntushelo, K. (2021) Correlations between root metabolomics and bacterial community structures in the phragmites australis under acid mine drainage-Polluted wetland ecosystem. Curr. Microbiol. 79, 34 10.1007/s00284-021-02748-7 34962589 PMC8714630

[54] Li, X., Qu, C., Bian, Y., Gu, C., Jiang, X. and Song, Y. (2019) New insights into the responses of soil microorganisms to polycyclic aromatic hydrocarbon stress by combining enzyme activity and sequencing analysis with metabolomics. Environ. Pollut. 255, 113312 10.1016/j.envpol.2019.113312 31610503

[55] Neethu, C.S., Saravanakumar, C., Purvaja, R., Robin, R.S. and Ramesh, R. (2019) Oil-Spill triggered shift in indigenous microbial structure and functional dynamics in different marine environmental matrices. Sci. Rep. 9, 1354 10.1038/s41598-018-37903-x 30718727 PMC6361881

[56] Shah, R.M., Stephenson, S., Crosswell, J., Gorman, D., Hillyer, K.E., Palombo, E.A. et al. (2021) Omics-based ecosurveillance uncovers the influence of estuarine macrophytes on sediment microbial function and metabolic redundancy in a tropical ecosystem. Sci. Total Environ. 809, 151175 10.1016/j.scitotenv.2021.151175 34699819

[57] Borsetto, C., Raguideau, S., Travis, E., Kim, D.-W., Lee, D.-H., Bottrill, A. et al. (2021) Impact of sulfamethoxazole on a riverine microbiome. Water Res. 201, 117382 10.1016/j.watres.2021.117382 34225233

[58] Bergauer, K., Fernandez-Guerra, A., Garcia, J.A.L., Sprenger, R.R., Stepanauskas, R., Pachiadaki, M.G. et al. (2018) Organic matter processing by microbial communities throughout the atlantic water column as revealed by metaproteomics. Proc. Natl Acad. Sci. U.S.A. 115, E400–e408 10.1073/pnas.1708779115 29255014 PMC5776962

[59] Starke, R., Bastida, F., Abadía, J., García, C., Nicolás, E. and Jehmlich, N. (2017) Ecological and functional adaptations to water management in a semiarid agroecosystem: a soil metaproteomics approach. Sci. Rep. 7, 10221 10.1038/s41598-017-09973-w 28860535 PMC5579227

[60] Taubert, M., Grob, C., Crombie, A., Howat, A.M., Burns, O.J., Weber, M. et al. (2019) Communal metabolism by methylococcaceae and methylophilaceae is driving rapid aerobic methane oxidation in sediments of a shallow seep near elba, Italy. Environ. Microbiol. 21, 3780–3795 10.1111/1462-2920.14728 31267680

[61] Liang, R., Li, Z., Lau Vetter, M.C.Y., Vishnivetskaya, T.A., Zanina, O.G., Lloyd, K.G. et al. (2021) Genomic reconstruction of fossil and living microorganisms in ancient siberian permafrost. Microbiome 9, 110 10.1186/s40168-021-01057-2 34001281 PMC8130349

[62] Li, M., Qian, W.-J., Gao, Y., Shi, L. and Liu, C. (2017) Functional enzyme-Based approach for linking microbial community functions with biogeochemical process kinetics. Environ. Sci. Technol. 51, 11848–11857 10.1021/acs.est.7b03158 28891285

[63] Yu, K., Yi, S., Li, B., Guo, F., Peng, X., Wang, Z. et al. (2019) An integrated meta-omics approach reveals substrates involved in synergistic interactions in a bisphenol A (BPA)-degrading microbial community. Microbiome 7, 16–16 10.1186/s40168-019-0634-5 30728080 PMC6366072

[64] Karthikeyan, S., Kim, M., Heritier-Robbins, P., Hatt, J.K., Spain, J.C., Overholt, W.A. et al. (2020) Integrated omics elucidate the mechanisms driving the rapid biodegradation of deepwater horizon Oil in intertidal sediments undergoing oxic-Anoxic cycles. Environ. Sci. Technol. 54, 10088–10099 10.1021/acs.est.0c02834 32667785

[65] Fernandes, M.L.P., Bastida, F., Jehmlich, N., Martinović, T., Větrovský, T., Baldrian, P. et al. (2022) Functional soil mycobiome across ecosystems. J. Proteomics 252, 104428 10.1016/j.jprot.2021.104428 34818587

[66] Laroche, O., Wood, S.A., Tremblay, L.A., Ellis, J.I., Lear, G. and Pochon, X. (2018) A cross-taxa study using environmental DNA/RNA metabarcoding to measure biological impacts of offshore oil and gas drilling and production operations. Mar. Pollut. Bull. 127, 97–107 10.1016/j.marpolbul.2017.11.042 29475721

[67] Bastida, F., Jehmlich, N., Lima, K., Morris, B.E.L., Richnow, H.H., Hernández, T. et al. (2016) The ecological and physiological responses of the microbial community from a semiarid soil to hydrocarbon contamination and its bioremediation using compost amendment. J. Proteomics 135, 162–169 10.1016/j.jprot.2015.07.023 26225916

[68] Benndorf, D., Balcke, G.U., Harms, H. and von Bergen, M. (2007) Functional metaproteome analysis of protein extracts from contaminated soil and groundwater. ISME J. 1, 224–234 10.1038/ismej.2007.39 18043633

[69] Gouveia, D., Almunia, C., Cogne, Y., Pible, O., Degli-Esposti, D., Salvador, A. et al. (2019) Ecotoxicoproteomics: a decade of progress in our understanding of anthropogenic impact on the environment. J. Proteomics 198, 66–77 10.1016/j.jprot.2018.12.001 30529745

[70] Gandar, A., Laffaille, P., Marty-Gasset, N., Viala, D., Molette, C. and Jean, S. (2017) Proteome response of fish under multiple stress exposure: effects of pesticide mixtures and temperature increase. Aquat. Toxicol. 184, 61–77 10.1016/j.aquatox.2017.01.004 28109940

[71] Simmons, D.B.D. and Sherry, J.P. (2016) Plasma proteome profiles of white sucker (Catostomus commersonii) from the Athabasca river within the oil sands deposit. Comp. Biochem. Physiol. Part D Genomics Proteomics 19, 181–189 10.1016/j.cbd.2016.03.003 27013027

[72] McCuaig, L.M., Martyniuk, C.J. and Marlatt, V.L. (2020) Morphometric and proteomic responses of early-life stage rainbow trout (Oncorhynchus mykiss) to the aquatic herbicide diquat dibromide. Aquat. Toxicol. 222, 105446 10.1016/j.aquatox.2020.105446 32092595

[73] Bertrand, L., Monferrán, M.V., Mouneyrac, C. and Amé, M.V. (2018) Native crustacean species as a bioindicator of freshwater ecosystem pollution: a multivariate and integrative study of multi-biomarker response in active river monitoring. Chemosphere 206, 265–277 10.1016/j.chemosphere.2018.05.002 29753289

[74] Muralidharan, S., Thompson, E., Raftos, D., Birch, G. and Haynes, P.A. (2012) Quantitative proteomics of heavy metal stress responses in sydney rock oysters. Proteomics 12, 906–921 10.1002/pmic.201100417 22539440

[75] Simões, T., Novais, S.C., Natal-da-Luz, T., Devreese, B., de Boer, T., Roelofs, D. et al. (2018) An integrative omics approach to unravel toxicity mechanisms of environmental chemicals: effects of a formulated herbicide. Sci. Rep. 8, 11376 10.1038/s41598-018-29662-6 30054531 PMC6063884

[76] Starke, R., Jehmlich, N. and Bastida, F. (2019) Using proteins to study how microbes contribute to soil ecosystem services: the current state and future perspectives of soil metaproteomics. J. Proteomics 198, 50–58 10.1016/j.jprot.2018.11.011 30445181

[77] Jouffret, V., Miotello, G., Culotta, K., Ayrault, S., Pible, O. and Armengaud, J. (2021) Increasing the power of interpretation for soil metaproteomics data. Microbiome 9, 195 10.1186/s40168-021-01139-1 34587999 PMC8482631

[78] Müller, J.B., Geyer, P.E., Colaço, A.R., Treit, P.V., Strauss, M.T., Oroshi, M. et al. (2020) The proteome landscape of the kingdoms of life. Nature 582, 592–596 10.1038/s41586-020-2402-x 32555458

[79] Keiblinger, K.M. and Riedel, K. (2018) Sample preparation for metaproteome analyses of soil and leaf litter. Methods Mol. Biol. 1841, 303–318 10.1007/978-1-4939-8695-8_21 30259495

[80] Chourey, K. and Hettich, R.L. (2018) Utilization of a detergent-Based method for direct microbial cellular lysis/Proteome extraction from soil samples for metaproteomics studies. Methods Mol. Biol. 1841, 293–302 10.1007/978-1-4939-8695-8_20 30259494

[81] Thorn, C.E., Bergesch, C., Joyce, A., Sambrano, G., McDonnell, K., Brennan, F. et al. (2019) A robust, cost-effective method for DNA, RNA and protein co-extraction from soil, other complex microbiomes and pure cultures. Mol. Ecol. Resour. 19, 439–455 10.1111/1755-0998.12979 30565880

[82] Wöhlbrand, L., Feenders, C., Nachbaur, J., Freund, H., Engelen, B., Wilkes, H. et al. (2017) Impact of extraction methods on the detectable protein complement of metaproteomic analyses of marine sediments. Proteomics 17. 10.1002/pmic.201700241 29027362

[83] Zou, T., Liang, Y.Q., Liao, X., Chen, X.F., Wang, T., Song, Y. et al. (2021) Metabolomics reveals the reproductive abnormality in female zebrafish exposed to environmentally relevant levels of climbazole. Environ. Pollut. 275, 116665 10.1016/j.envpol.2021.116665 33581626

[84] Huang, W., Wang, X., Chen, D., Xu, E.G. Luo, X., Zeng, J. et al. (2021) Toxicity mechanisms of polystyrene microplastics in marine mussels revealed by high-coverage quantitative metabolomics using chemical isotope labeling liquid chromatography mass spectrometry. J. Hazard. Mater. 417, 126003 10.1016/j.jhazmat.2021.126003 33992921

[85] Dumas, T., Bonnefille, B., Gomez, E., Boccard, J., Castro, N.A., Fenet, H. et al. (2020) Metabolomics approach reveals disruption of metabolic pathways in the marine bivalve mytilus galloprovincialis exposed to a WWTP effluent extract. Sci. Total Environ. 712, 136551 10.1016/j.scitotenv.2020.136551 31945539

[86] Gyawali, P., Karpe, A.V., Hillyer, K.E., Nguyen, T.V., Hewitt, J. and Beale, D.J. (2021) A multi-platform metabolomics approach to identify possible biomarkers for human faecal contamination in greenshell™ mussels (Perna canaliculus). Sci. Total Environ. 771, 145363 10.1016/j.scitotenv.2021.145363 33736167

[87] Jeong, T.Y. and Simpson, M.J. (2019) Daphnia magna metabolic profiling as a promising water quality parameter for the biological early warning system. Water Res. 166, 115033 10.1016/j.watres.2019.115033 31505309

[88] Vandenbrouck, T., Jones, O.A.H., Dom, N., Griffin, J.L. and De Coen, W. (2010) Mixtures of similarly acting compounds in daphnia magna: from gene to metabolite and beyond. Environ. Int. 36, 254–268 10.1016/j.envint.2009.12.006 20117838

[89] Kikuchi, J., Ito, K. and Date, Y. (2018) Environmental metabolomics with data science for investigating ecosystem homeostasis. Prog. Nucl. Magn. Reson. Spectrosc. 104, 56–88 10.1016/j.pnmrs.2017.11.003 29405981

[90] Peng, J., Guo, J., Lei, Y., Mo, J., Sun, H. and Song, J. (2021) Integrative analyses of transcriptomics and metabolomics in raphidocelis subcapitata treated with clarithromycin. Chemosphere 266, 128933 10.1016/j.chemosphere.2020.128933 33223212

[91] Shah, R.M., Hillyer, K.E., Stephenson, S., Crosswell, J., Karpe, A.V., Palombo, E.A. et al. (2021) Functional analysis of pristine estuarine marine sediments. Sci. Total Environ. 781, 146526 10.1016/j.scitotenv.2021.146526 33798899

[92] Jones, O.A.H., Lear, G., Welji, A.M., Collins, G. and Quince, C. (2016) Community Metabolomics in Environmental Microbiology. In Microbial Metabolomics: Applications in Clinical, Environmental, and Industrial Microbiology (Beale, D.J., Kouremenos, K.A. and Palombo, E.A., eds), pp. 199–224, Springer International Publishing, Cham

[93] Jones, O.A.H., Sdepanian, S., Lofts, S., Svendsen, C., Spurgeon, D.J., Maguire, M.L. et al. (2014) Metabolomic analysis of soil communities can be used for pollution assessment. Environ. Toxicol. Chem. 33, 61–64 10.1002/etc.2418 24122881

[94] Beale, D.J., Barratt, R., Marlow, D.R., Dunn, M.S., Palombo, E.A., Morrison, P.D. et al. (2013) Application of metabolomics to understanding biofilms in water distribution systems: a pilot study. Biofouling 29, 283–294 10.1080/08927014.2013.772140 23458161

[95] Malinowska, J.M., Palosaari, T., Sund, J., Carpi, D., Bouhifd, M., Weber, R.J.M. et al. (2022) Integrating in vitro metabolomics with a 96-well high-throughput screening platform. Metabolomics 18, 11 10.1007/s11306-021-01867-3 35000038 PMC8743266

[96] Yin, X., Altman, T., Rutherford, E., West, K.A., Wu, Y., Choi, J. et al. (2020) A comparative evaluation of tools to predict metabolite profiles from microbiome sequencing data. Front. Microbiol. 11, 595910 10.3389/fmicb.2020.595910 33343536 PMC7746778

[97] Hua, Q., Adamovsky, O., Vespalcova, H., Boyda, J., Schmidt, J.T., Kozuch, M. et al. (2021) Microbiome analysis and predicted relative metabolomic turnover suggest bacterial heme and selenium metabolism are altered in the gastrointestinal system of zebrafish (Danio rerio) exposed to the organochlorine dieldrin. Environ. Pollut. 268, 115715 10.1016/j.envpol.2020.115715 33069042

[98] Pinu, F.R., Beale, D.J., Paten, A.M., Kouremenos, K., Swarup, S., Schirra, H.J. et al. (2019) Systems biology and multi-Omics integration: viewpoints from the metabolomics research community. Metabolites 9, 76 10.3390/metabo9040076 PMC6523452 31003499

[99] Kosina, S.M., Greiner, A.M., Lau, R.K., Jenkins, S., Baran, R., Bowen, B.P. et al. (2018) Web of microbes (WoM): a curated microbial exometabolomics database for linking chemistry and microbes. BMC Microbiol. 18, 115 10.1186/s12866-018-1256-y 30208844 PMC6134592

[100] Noecker, C., Eng, A., Muller, E. and Borenstein, E. (2022) MIMOSA2: a metabolic network-based tool for inferring mechanism-supported relationships in microbiome-metabolome data. Bioinformatics 38, 1615–1623 10.1093/bioinformatics/btac003 PMC8896604 34999748

[101] Mallick, H., Franzosa, E.A., McLver, L.J., Banerjee, S., Sirota-Madi, A., Kostic, A.D. et al. (2019) Predictive metabolomic profiling of microbial communities using amplicon or metagenomic sequences. Nat. Commun. 10, 3136 10.1038/s41467-019-10927-1 31316056 PMC6637180

[102] Chong, J., Liu, P., Zhou, G. and Xia, J. (2020) Using microbiomeAnalyst for comprehensive statistical, functional, and meta-analysis of microbiome data. Nat. Protoc. 15, 799–821 10.1038/s41596-019-0264-1 31942082

[103] Griss, J., Viteri, G., Sidiropoulos, K., Nguyen, V., Fabregat, A. and Hermjakob, H. (2020) ReactomeGSA - efficient multi-Omics comparative pathway analysis. Mol. Cell. Proteomics 19, 2115–2125 10.1074/mcp.TIR120.002155 32907876 PMC7710148

[104] Hernández-de-Diego, R., Tarazona, S., Martínez-Mira, C., Balzano-Nogueira, L., Furió-Tarí, P., Pappas, Jr, G.J. et al. (2018) Paintomics 3: a web resource for the pathway analysis and visualization of multi-omics data. Nucleic Acids Res. 46, W503–w509 10.1093/nar/gky466 29800320 PMC6030972

[105] Rohart, F., Gautier, B., Singh, A. and Lê Cao, K.-A. (2017) Mixomics: an R package for ‘omics feature selection and multiple data integration. PLoS Comput. Biol. 13, e1005752–e1005752 10.1371/journal.pcbi.1005752 29099853 PMC5687754

[106] Zhou, G., Ewald, J. and Xia, J. (2021) Omicsanalyst: a comprehensive web-based platform for visual analytics of multi-omics data. Nucleic Acids Res. 49, W476–W482 10.1093/nar/gkab394 34019646 PMC8262745

[107] Zhou, G. and Xia, J. (2019) Using omicsNet for network integration and 3D visualization. Curr. Protoc. Bioinformatics 65, e69 10.1002/cpbi.69 30556956

[108] Mancia, A. (2018) New technologies for monitoring marine mammal health. Mar. Mamm. Ecotoxicol., 291–320 10.1016/B978-0-12-812144-3.00011-5

[109] Jubeaux, G., Audouard-Combe, F., Simon, R., Tutundjian, R., Salvador, A., Geffard, O. et al. (2012) Vitellogenin-like proteins among invertebrate species diversity: potential of proteomic mass spectrometry for biomarker development. Environ. Sci. Technol. 46, 6315–6323 10.1021/es300550h 22578134

[110] Dias, D.A., Jones, O.A.H., Beale, D.J., Boughton, B.A., Benheim, D., Kouremenos, K.A. et al. (2016) Current and future perspectives on the structural identification of small molecules in biological systems. Metabolites 6, 46 10.3390/metabo6040046 PMC5192452 27983674

[111] Jones, O.A.H. (2018) Illuminating the dark metabolome to advance the molecular characterisation of biological systems. Metabolomics 14, 101 10.1007/s11306-018-1396-y 30830382

[112] Wishart, D.S., Feunang, Y.D., Marcu, A., Guo, A.C., Liang, K., Vázquez-Fresno, R. et al. (2017) HMDB 4.0: the human metabolome database for 2018. Nucleic Acids Res. 46, D608–D617 10.1093/nar/gkx1089 PMC5753273 29140435

[113] Xue, J., Guijas, C., Benton, H.P., Warth, B. and Siuzdak, G. (2020) METLIN MS2 molecular standards database: a broad chemical and biological resource. Nat. Methods 17, 953–954 10.1038/s41592-020-0942-5 32839599 PMC8802982

[114] Wishart, D., Arndt, D., Pon, A., Sajed, T., Guo, A.C., Djoumbou, Y. et al. (2015) T3DB: the toxic exposome database. Nucleic Acids Res. 43, D928–D934 10.1093/nar/gku1004 25378312 PMC4383875

[115] Wishart, D.S., Knox, C., Guo, A.C., Cheng, D., Shrivastava, S., Tzur, D. et al. (2007) Drugbank: a knowledgebase for drugs, drug actions and drug targets. Nucleic Acids Res. 36, D901–D906 10.1093/nar/gkm958 18048412 PMC2238889

[116] Ulrich, E.M., Sobus, J.R., Grulke, C.M., Richard, A.M., Newton, S.R., Strynar, M.J. et al. (2019) EPA's non-targeted analysis collaborative trial (ENTACT): genesis, design, and initial findings. Anal. Bioanal. Chem. 411, 853–866 10.1007/s00216-018-1435-6 30519961 PMC7477838

[117] Gillet, L.C., Navarro, P., Tate, S., Röst, H., Selevsek, N., Reiter, L. et al. (2012) Targeted data extraction of the MS/MS spectra generated by data-independent acquisition: a new concept for consistent and accurate proteome analysis*. Mol. Cell. Proteomics 11, O111.016717 10.1074/mcp.O111.016717 PMC3433915 22261725

[118] Long, S., Yang, Y., Shen, C., Wang, Y., Deng, A., Qin, Q. et al. (2020) Metaproteomics characterizes human gut microbiome function in colorectal cancer. NPJ Biofilms Microbiomes 6, 14 10.1038/s41522-020-0123-4 32210237 PMC7093434

[119] Demichev, V., Messner, C.B., Vernardis, S.I., Lilley, K.S. and Ralser, M. (2020) DIA-NN: neural networks and interference correction enable deep proteome coverage in high throughput. Nat. Methods 17, 41–44 10.1038/s41592-019-0638-x 31768060 PMC6949130

[120] Gessulat, S., Schmidt, T., Zolg, D.P., Samaras, P., Schnatbaum, K., Zerweck, J. et al. (2019) Prosit: proteome-wide prediction of peptide tandem mass spectra by deep learning. Nat. Methods 16, 509–518 10.1038/s41592-019-0426-7 31133760

